# Rare Multidrug-Resistant Pulmonary Nocardiosis in AIDS

**DOI:** 10.7759/cureus.1839

**Published:** 2017-11-11

**Authors:** Cherry O Onaiwu, Manasa Velagapudi, Luay Sarsam, Lindsay Utley, Lauren Bricker, Venkata Sunil Bendi, Renuga Vivekanandan

**Affiliations:** 1 Department of Internal Medicine, Creighton University Medical Center; 2 Department of Infectious Diseases, Creighton University Medical Center; 3 Department of Pharmacology, Creighton University Medical Center; 4 Department of Pharmacology and Experimental Neuroscience, University of Nebraska Medical Center

**Keywords:** pulmonary nocardia, nocardia farcinica, aids

## Abstract

Nocardiosis is an opportunistic infection in patients with depressed cell-mediated immunity. Inhalation is the primary route for exposure via dust particles. Patients with acquired immune deficiency syndrome (AIDS) are at increased risk of disseminated disease. A challenge in the diagnosis of pulmonary nocardiosis is that it can mimic other pulmonary diseases. Nocardia farcinica tends to be a more virulent, multidrug-resistant strain with an increased tendency to disseminate. This report describes a 64-year-old man with AIDS found to have pulmonary nocardiosis that did not respond to standard antibiotic therapy. Further evaluation revealed the virulent, multidrug-resistant Nocardia farcinica species. Targeted antibiotic therapy was initiated, after which the patient had an improvement in pulmonary symptoms. It is important to suspect pulmonary nocardiosis in immunocompromised patients who fail to respond to standard antimicrobial therapy. Susceptibilities should be obtained so that appropriate therapy can be promptly initiated as Nocardia farcinica is highly resistant to multiple antimicrobials.

## Introduction

Nocardia is an aerobic, gram-positive, beaded, branching, filamentous bacteria found in soil, decomposing vegetation, fresh water, or salt water [1]. It usually manifests as an opportunistic infection that can cause localized or disseminated disease involving the central nervous system (CNS), bones, joints, liver, and pericardium [2]. Transmission is through the inhalation or direct inoculation of spores and inhalation is the primary route for exposure usually via dust particles [1,3]. Despite its low prevalence, when nocardiosis occurs, it is associated with high mortality in acquired immune deficiency syndrome (AIDS) patients.

## Case presentation

A 64-year-old African American man presented after being found minimally responsive by family. Prior to arrival, emergency medical services (EMS) noted that the patient was hypoglycemic and administered intravenous (IV) dextrose after which the patient became more alert. On arrival, his review of systems was positive for a cough without hemoptysis, dyspnea, a several-month history of weakness, general malaise, intermittent nausea and vomiting, dizziness, fatigue, anorexia, and an unintentional 50 lb weight loss over the past six months. 

The patient had a past medical history of depression, AIDS, chronic hepatitis C, esophageal candidiasis, herpes simplex, nonobstructive coronary artery disease, and Takotsubo cardiomyopathy. Social history included polysubstance abuse. Current home medications included oral antifungal therapy, antihypertensives, and antidepressants.

On presentation, vital signs were significant for hypothermia with a temperature of 91 degrees Fahrenheit, hypotension with a blood pressure of 85/57, and an oxygen saturation of 93% on room air. Heart rate and respiratory rates were within normal limits. Physical exam demonstrated cachexia, altered mental status, delirium, coarse breath sounds, and bilateral lower extremity edema. The patient quickly deteriorated and became hypoxic. He was intubated, aggressively fluid resuscitated, rewarmed, and started on empiric antibiotic treatment with vancomycin, piperacillin-tazobactam, and levofloxacin. Hypotension persisted, and vasopressors were initiated for septic shock. 

**Figure 1:**
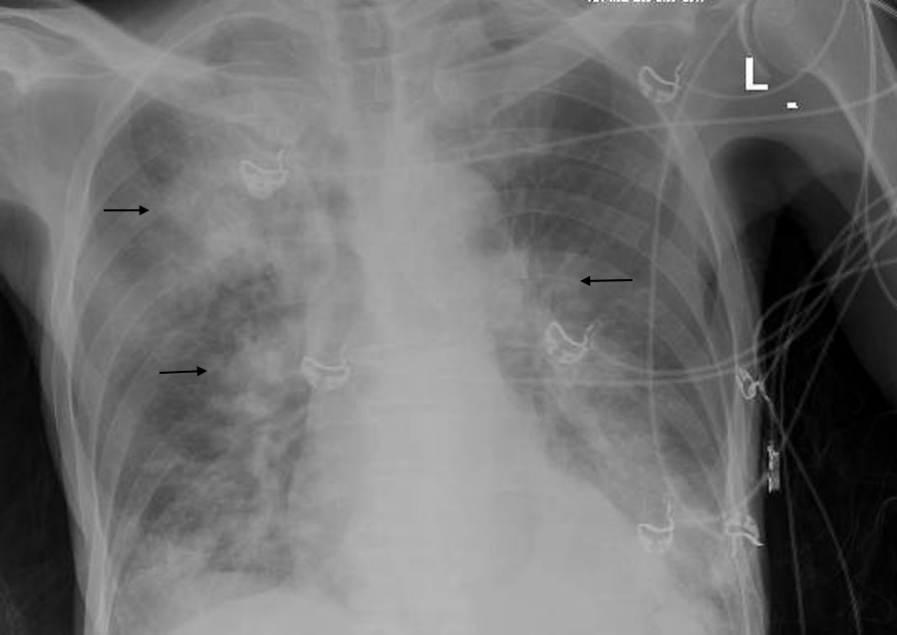
Chest radiograph showing diffuse opacification and emphysematous changes

**Figure 2:**
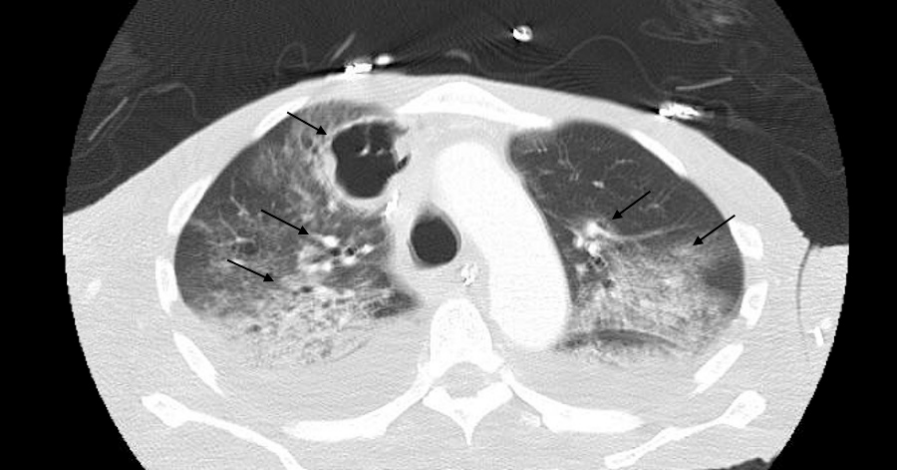
CT chest showing diffuse ground glass opacities

Initial diagnostic studies were significant for pancytopenia, lactic acidosis, and transaminitis. The patient had a CD4 count of 58 cells/cubic millimeter. A chest radiograph showed diffuse opacification and emphysematous changes (Figure 1). A computed tomography (CT) scan of the chest showed a right, upper lobe, lobulated, thick wall cavitary lesion and diffuse ground-glass opacities, which confirmed multifocal pneumonia (Figure 2). His antiretroviral therapy of emtricitabine/tenofovir and dolutegravir was continued, and he was started on IV fluconazole for his esophageal candidiasis. In addition, therapeutic treatment with trimethoprim/sulfamethoxazole (TMP/SMX) for presumptive Pneumocystis jirovecii pneumonia was initiated. Further diagnostic studies revealed Klebsiella bacteremia, positive Toxoplasma IgG, and positive 1,3-beta-D-glucan.

Ophthalmology was consulted and ruled out ocular toxoplasmosis and cytomegalovirus retinitis. Despite appropriate antibiotic and antifungal therapy for several days, his oxygenation did not improve and repeat chest imaging demonstrated persistent and worsening pneumonia. Other infectious etiologies, including Mycobacterium tuberculosis, Pneumococcus, Legionella, and Cryptococcus were ruled out. Blood cultures revealed branching gram-positive rods, which were identified as Nocardia by acid-fast staining. Bronchoscopy was done and aspirate cultures revealed Nocardia species.

Vancomycin and levofloxacin were discontinued and ceftriaxone was started, which, in addition to the TMP/SMX he had already been receiving, should have offered double coverage. He did not improve with this regimen and, therefore, species identification was requested and revealed Nocardia farcinica species. The isolate was susceptible only to amikacin, ciprofloxacin, imipenem/cilastatin, linezolid, moxifloxacin, penicillin, and trimethoprim/sulfamethoxazole, was resistant to cefepime, ceftriaxone, clarithromycin, and tobramycin, and was intermediately susceptible to doxycycline. Ceftriaxone was, hence, changed to moxifloxacin. The patient had an improvement in infiltrates and oxygenation with the final antibiotic regimen consisting of TMP/SMX and moxifloxacin.

## Discussion

Epidemiology

With over 50 species reported to date, Nocardia has an incidence of up to 1000 new cases per year [1]. Pulmonary nocardiosis has a low reported prevalence of 0.3% in AIDS [2]. This rarity can be due to its nonspecific clinical and radiologic presentation, the difficult microbiological isolation that results in underdiagnoses, the frequency of coinfections, and the widespread use of TMP/SMX prophylaxis in the United States [2]. With the advent of highly potent antiretroviral therapy, the incidence of Nocardiosis further declined, similar to other opportunistic infections in AIDS. Fourteen to 27% of *Nocardia* cases belong to the farcinica species. Nocardia farcinica is typically a more virulent, multidrug-resistant strain with an increased tendency to disseminate [4].

Risk factors

Nocardia usually manifests as an opportunistic infection in immunocompromised hosts with depressed cell-mediated immunity. Immunocompetent patients can also be affected, with some reports showing up to one-third of nocardiosis cases occurring in the immunocompetent [1]. Some common impacted immunosuppressive states reported included lymphoma, other malignancies, transplant recipients, long-term steroid use, and AIDS [5]. Nocardiosis in AIDS patients is more common at CD4 counts less than 200 cells/cubic millimeter and in those not on antiretroviral therapy. Injection drug use also appears to increase the risk of nocardiosis [2]. The patient had AIDS with a CD4 count of 58 cells/cubic millimeter and a history of IV drug use, making him more susceptible to the Nocardia infection.

Clinical features of pulmonary nocardiosis

Pulmonary nocardiosis is the most common clinical presentation of nocardiosis (>40% of reported cases) because inhalation is the primary route of bacterial exposure [6]. It can manifest as an acute, subacute, or chronic infection with remissions and exacerbations. The patient presented with fever, fatigue, cough, dyspnea, and unintentional weight loss. Cough (productive or nonproductive) was the most common presenting symptom of pulmonary nocardiosis followed by fever, dyspnea, chest pain, hemoptysis, night sweats, weight loss, and progressive fatigue. In patients with AIDS, nocardiosis presents as alveolar infiltrates that progress during therapy rather than as cavitary lesions [7]. Extrapulmonary involvement is very common, with the central nervous system (CNS) being the most common site of occurrence with up to 44% reported in one series [6]. Isolated CNS disease, however, may occur. 

Diagnosis of pulmonary nocardiosis

Nocardiosis poses a diagnostic challenge due to the difficulty in isolating the organism, nonspecific presentation, and the debilitating course that mimics many other infections. It is a very slow-growing, aerobic, gram-positive bacteria in respiratory specimens, and without selective media, its growth is obscured by other rapidly growing bacteria. The gram stain of the respiratory specimens demonstrating filamentous, beaded, gram-positive bacteria followed by auramine O staining with modified Ziehl-Neelsen staining has been shown to aid in timely diagnosis. Samples taken by both fine needle aspiration cytology and microbial cultures from sputum or bronchial washing are often required to get a diagnosis [8]. The Nocardia species in the patient was identified by the above-mentioned methods, including a gram stain of respiratory specimens and the bronchial aspirate. 

The isolation of Nocardia requires selective media and Nocardia farcinica is typically known to cause the opacification of Middlebrook agar. The identification of Nocardia from an immunocompromised patient should never be ignored, especially if any abnormal clinical or radiologic pulmonary findings are present [1]. On chest radiography and computed tomography (CT), the patient had diffuse ground-glass opacification, emphysematous changes, and a cavitary lesion. CT does not provide a definite diagnosis but does provide typical findings of multiple pulmonary nodules (often cavitary), consolidation, or bronchiectasis. CNS imaging, preferably magnetic resonance imaging (MRI), should be considered for patients with any adverse neurologic symptoms, severe pulmonary nocardiosis, or significant immunosuppression [1]. The patient did have an MRI of the brain, which was insignificant for CNS involvement. 

Nocardiosis treatment

Due to an absence of randomized controlled trials, the treatment of pulmonary nocardiosis is guided by retrospective studies and in vitro susceptibility patterns [1,4]. Sulfonamides have been the drugs of choice to treat nocardiosis since 1944 [9]. TMP/SMX is commonly used as part of empiric therapy at 5 to 10 mg/kg/day in divided doses, based on the TMP component [1,9]. However, some Nocardia isolates have been classified as resistant to TMP/SMX [4]. In addition to high disease severity, potential resistance to TMP/SMX justifies combination therapy. Empiric treatment should consist of at least two antibiotics due to variable resistance rates of Nocardia. Combination therapy should be continued until the species is known and the patient’s clinical status improves [1,9]. The patient's clinical status did not initially improve with TMP/SMX and ceftriaxone combination therapy. Identification of the Nocardia species, followed by antibiotic susceptibilities, is essential to further direct treatment.

Nocardia farcinica is one of the species known to have multidrug resistance [1,9]. Nocardia farcinica is generally resistant to all other aminoglycosides (except amikacin), ampicillin, ceftriaxone, and clarithromycin [1,9]. Based on susceptibility patterns, the following antibiotics could be used in conjunction with TMP/SMX: amikacin, imipenem, fluoroquinolones, and linezolid for the treatment of Nocardia farcinica [1,9]. The patient's isolate was susceptible only to amikacin, ciprofloxacin, imipenem/cilastatin, linezolid, moxifloxacin, penicillin, and trimethoprim/sulfamethoxazole and resistant to cefepime, ceftriaxone, clarithromycin, and tobramycin, and intermediately susceptible to doxycycline. He was ultimately treated with TMP/SMX and moxifloxacin with improvement in his symptoms.

The length of pulmonary nocardiosis treatment depends on the immune status of the patient. The duration of therapy for immunosuppressed individuals is at least 12 months to prevent relapse [1,9-10]. Combination therapy should be initiated via the IV route. After three to six weeks of IV therapy with clinical improvement, some reports state that switching to oral therapy may be appropriate [10]. Suggested oral antibiotics, if susceptible, include TMP/SMX, minocycline, fluoroquinolones, linezolid, and amoxicillin-clavulanate [10].

## Conclusions

The patient had multiple risk factors, deeming him more susceptible to the Nocardia infection, including his immunocompromised state, AIDS with a CD4 count less than 200 cells/cubic millimeter, and a history of IV drug use, among others. Furthermore, due to the nonspecific presentation of pulmonary nocardiosis and his confounding comorbidities, diagnosis in the patient was not initially obvious. His lack of improvement with standard therapy, TMP/SMX, is what prompted us to evaluate further, which eventually revealed the resistant Nocardia farcinica strain. Once susceptibilities revealed multidrug resistance, his medication regimen was adjusted to TMP/SMX and moxifloxacin with an improvement in his symptoms. 

Pulmonary nocardiosis is a rare occurrence in AIDS patients, with a reported prevalence of 0.3% in the United States. It is often misdiagnosed, as symptoms are nonspecific and may mimic other, more common pulmonary infections. Indolent pulmonary disease in the setting of compromised cellular immunity like AIDS should raise suspicion for pulmonary nocardiosis. Failure to initiate appropriate antimicrobial therapy promptly, delay in diagnosis, and disseminated disease are associated with poor prognosis. This case highlights the importance of suspecting pulmonary nocardiosis in AIDS patients who fail to respond to standard antimicrobial therapy, as with our patient, and the importance of obtaining species identification and susceptibility testing.
